# Single-Lead ECG Arrhythmia Classification Based on Peak-Enhanced Attention Network and Quality-Aware GAN Data Augmentation Framework

**DOI:** 10.3390/s26123852

**Published:** 2026-06-17

**Authors:** Yaoyu Zhang, Yi Xia

**Affiliations:** The School of Electrical Engineering and Automation, Anhui University, Hefei 230601, China; z24301058@stu.ahu.edu.cn

**Keywords:** atrial fibrillation, electrocardiogram (ECG), generative adversarial network, attention mechanism, single-lead ECG

## Abstract

Single-lead electrocardiogram (ECG) is widely used in wearable devices for atrial fibrillation (AF) screening. Nevertheless, subtle pathological characteristics like P-waves and f-waves in practical signals are vulnerable to noise contamination. Meanwhile, the scarcity of high-quality annotated abnormal data instances leads to severe class imbalance. To mitigate these issues, we present an end-to-end framework designed for arrhythmia diagnosis using single-lead ECG signals, which integrates quality-aware data augmentation with a Peak-Enhanced attention mechanism. First, to mitigate the problem of data imbalance, a Quality-Aware Generative Adversarial Network (QA-GAN) is designed. This network integrates a signal quality evaluation module based on signal kurtosis, together with a dynamic soft-label training scheme, guiding the generator to prioritize learning high-quality morphological features, thereby synthesizing high-fidelity minority class samples. Second, to accurately capture subtle pathological features in electrocardiograms, a Peak-Enhanced Attention Convolutional Network (PEAC-Net) classification model is proposed. This model incorporates a Peak-Enhanced Attention (PE-Att) module, which employs learnable derivative convolutional kernels to precisely identify the transition points in the ECG signal. Furthermore, by integrating one-dimensional multi-scale dilated convolution (DSGC1D) with bidirectional LSTM, the model achieves effective capturing of both fine-grained local morphological features and long-range global rhythm patterns. Experimental results on the PhysioNet 2017 dataset indicate that the presented model attains an accuracy of 0.902 and a macro-F1 score of 0.880, respectively, outperforming other state-of-the-art models and also exhibiting robust data adaptability on the MIT-BIH dataset.

## 1. Introduction

According to statistics from the World Health Organization [1], cardiovascular diseases (CVDs) are still the primary cause of mortality worldwide, responsible for roughly 17.7 million fatalities annually. Among various cardiac abnormalities, as the most common type of persistent cardiac arrhythmia, atrial fibrillation (AF) places a heavy burden on public health due to its often asymptomatic nature, unpredictability, and high risk of triggering stroke and heart failure [2]. Consequently, developing efficient and accurate techniques for AF screening and monitoring holds significant clinical importance.

Traditional diagnosis of atrial fibrillation primarily relies on standard 12-lead electrocardiograms (ECGs). Although this method is the clinical gold standard, its reliance on complex equipment, specialized operation, and inability to support long-term continuous monitoring make it difficult to capture the fleeting pathological characteristics of paroxysmal atrial fibrillation. With the rise in mobile health technologies, wearable devices based on single-lead ECG have emerged as an important tool for AF screening, leveraging their portability and advantages in at-home monitoring. However, applying single-lead devices to complex real-world scenarios still faces several challenges. The scarcity of high-quality annotated data, particularly the limited availability of AF samples, leads to severe data imbalance, causing models to be highly prone to bias toward the majority class (normal sinus rhythm). On the other hand, subtle features are prone to be neglected. Key pathological characteristics in single-lead signals, such as P-waves and f-waves, not only exhibit low amplitude but also inherently manifest as subtle inflection points or rapid transitions in the signal. Under interference from baseline drift and motion artifacts, common convolution operations often struggle to distinguish these subtle morphological variations from high-frequency noise. As a result, crucial diagnostic cues (e.g., the onset of P-waves or the oscillations of f-waves) tend to be smoothed out or overlooked during feature extraction.

Existing studies predominantly employ traditional approaches, such as oversampling or the Synthetic Minority Over-sampling Technique (SMOTE), to address the issue of data imbalance [3]. Nevertheless, linear interpolation approaches like SMOTE are inadequate to effectively model the intricate nonlinear dynamics of ECG signals. In recent years, although Generative Adversarial Networks (GANs) have exhibited substantial potential in data synthesis, traditional GAN models often tend to fit the noise distribution when processing noisy ECG datasets. This results in generated samples containing artifacts and lacking clinical authenticity. However, despite the notable progress of deep learning in ECG analysis, certain morphological features (e.g., P-wave onset/offset points and R-wave peaks) remain difficult to extract effectively due to noise interference or the subtle manifestation at the early stage of the disease. Consequently, the identification of such abnormal ECG signals still faces challenges.

In response to the above challenges, a single-lead ECG analysis framework is proposed in this study, which integrates quality-aware data augmentation with a peak-enhanced attention mechanism. First, to address the issues of data imbalance and noise interference, we designed a Quality-Aware Generative Adversarial Network (QA-GAN). Unlike traditional data augmentation methods, QA-GAN incorporates a signal quality assessment mechanism based on the kurtosis of ECG signals, leveraging the sparsity of core features in ECG signals for the quantification of the importance of QRS complexes. Building on this, we adopted a dynamic soft-label training strategy, which guides the generator to prioritize learning high-quality morphological features by reducing the weight of noisy samples, thereby synthesizing high-fidelity ECG data. Second, to improve the model’s ability to capture critical features for diagnosis, we propose the Peak-Enhanced Attention Convolutional Network (PEAC-Net). PEAC-Net incorporates learnable derivative convolutional kernels to construct a Peak-Enhanced Attention module, enabling it to better adapt to abrupt transitions (R-waves) and inflection points (P-waves/T-waves) in ECG signals. This achieves precise localization and enhancement of key peaks. Simultaneously, by integrating one-dimensional dilated separable group convolution (DSGC1D) with bidirectional Long Short-Term Memory networks (BiLSTM), the model can capture both fine-grained local morphological traits and long-range global temporal dependencies. This greatly improves the model’s capability in representing subtle local morphological changes and global abnormal rhythms in complex arrhythmic signals. The key contributions of this work are listed as follows:

1. QA-GAN is designed to address the bottleneck of severe training data imbalance. To tackle the scarcity of atrial fibrillation samples, we introduce a dynamic soft-label mechanism based on Kurtosis, guiding the generator to synthesize ECG samples with clear pathological details. This method effectively avoids noise fitting and thus reconstructs a class-balanced training set for boosting the model’s capability to detect minority class samples.
2. A classification network with peak-enhanced attention and multi-scale spatio-temporal modeling (PEAC-Net) is designed to strengthen the model’s proficiency in capturing diagnostically relevant features of atrial fibrillation. To compensate for the inadequate capacity of traditional deep learning models to capture subtle morphological features, we propose a Peak-Enhanced Attention (PE-Att) mechanism based on learnable derivative kernels. This module improves the model’s sensitivity to signal transition points and inflection points through the adaptive evolution of first-order and second-order derivative operators. By integrating one-dimensional dilated separable group convolution (DSGC1D) with bidirectional LSTM, the model improves both the capability of capturing local waveform details and the ability to characterize global long-range rhythms of the ECG signals. The results on the PhysioNet 2017 dataset demonstrate that our model is remarkably better than current methods.

## 2. Related Work

### 2.1. Data Augmentation for Imbalanced ECG Instances

ECG datasets often face the challenge of data imbalance, where the number of normal sinus rhythm recordings far exceeds that of arrhythmias such as atrial fibrillation. This long-tailed distribution often leads classifiers to favor the majority class, thereby reducing sensitivity in atrial fibrillation detection. To mitigate this issue, early research primarily relied on oversampling techniques. Fan et al. leveraged random oversampling techniques to boost the representation of minority class instances [4]. Similarly, Cao et al. employed random window resampling to balance the training set [5]. Other researchers applied SMOTE and its variants, generating new samples through linear interpolation [6]. However, such linear interpolation methods struggle to capture the complex nonlinear characteristics of ECG signals, often resulting in synthesized samples that lack physiological fidelity or merely replicate existing noise.

Recently, GANs have emerged as a powerful alternative for synthesizing realistic time-series data. Delaney et al. explored the use of various GAN architectures, including LSTM-based generators, to enhance the authenticity of signals [7]. Banerjee and Ghose also demonstrated the great potential of GANs in generating synthetic ECG waveforms to augment training datasets [8]. Despite these advancements, a significant issue often overlooked in most GAN models is how to construct an effective signal quality assessment mechanism to guide the synthesis of generated signals when these models are trained on practical ECG signals. Consequently, generators often learn and replicate the noise distribution present in real data, such as baseline drift and artifacts. Although recent work, such as that by Msigwa et al., has proposed combining clustering algorithms (DBSCAN) to filter outliers before training [9], an end-to-end quality-aware generation mechanism is still lacking to dynamically guide the generator in prioritizing high-quality morphological features of the ECG signals. In response to this challenge, a Quality-Aware Generative Adversarial Network (QA-GAN) is proposed in this study.

### 2.2. Deep Learning and Attention Mechanisms for Feature Extraction

In the past decades, the identification methods for single-lead ECG arrhythmias have evolved from handcrafted feature extraction towards deep learning techniques. Convolutional neural networks (CNNs) exhibit superior performance in capturing local morphological characteristics, while recurrent neural networks (RNNs), particularly LSTM networks, are extensively utilized to model the temporal dependencies and irregular RR intervals characteristic of atrial fibrillation (AF). Limam and Precioso introduced a hybrid architecture, i.e., a convolutional recurrent neural network, that leverages the complementary advantages of CNNs and RNNs for enhanced classification [10]. Xu et al. introduced a hybrid architecture that integrates CNN with bidirectional LSTM for ECG analysis, demonstrating that the fusion of morphological features and temporal features effectively improves the accuracy of rhythm classification [11]. These studies demonstrate that combining morphological and temporal features effectively improves rhythm classification accuracy. However, RNN-based networks still face challenges such as vanishing gradients and low parallel computing efficiency when processing extremely long sequences.

In recent years, attention mechanisms and Transformer architectures have become research hotspots due to their global modeling capabilities. Qiu et al. proposed the Spatio-Temporal Convolutional Transformer network (STCT), which leverages the Transformer to directly model long-range dependencies between heartbeats, overcoming the limitations of traditional convolutions constrained by receptive fields [12]. Building on this, Islam et al. further proposed CAT-Net, a deep hybrid network integrating convolution, attention, and Transformer. This model utilizes CNNs to extract local waveform characteristics and employs Transformers to model global contextual information, achieving outstanding performance in single-lead ECG classification [13]. In addition to architectural innovations, the fusion of features from different domains is also very important. For example, the Dual-Domain Attention Cascade Network (D2AFNet) was proposed by Zhang et al. [14]. This model leverages concurrent attention in time and frequency domains to strengthen the detection of AF-related frequency characteristics, notably f-waves.

In summary, although hybrid architectures (such as CNN-Transformer) and multi-domain attention networks have significantly improved detection accuracy by introducing global receptive fields, these general-purpose deep networks often lack customized designs tailored specifically to ECG pathological features. Conventional global attention mechanisms tend to capture prominent global rhythm abnormalities, while they often suffer from weight dispersion when dealing with subtle local morphological alterations in single-lead signals, such as f-wave oscillations or the absence of P-waves. This makes it difficult to achieve precise focus on critical pathological inflection points. Furthermore, existing research has largely focused on architectural stacking, while overlooking the important role that high-quality training data plays in model performance. Therefore, how to improve the ability to capture weak features by introducing effective attention mechanisms is still a key problem for ECG analysis. To this end, a peak-enhanced attention mechanism is proposed in this study.

## 3. Methods

### 3.1. Experimental Database

The primary dataset utilized in this study for model training, validation, and testing is the PhysioNet Challenge 2017 [15]. A total of 8528 single-lead ECG recordings are included in this dataset, which were sampled at 300 Hz and obtained using AliveCor devices. As shown in Table 1, the recordings were annotated by experts into four classes: normal sinus rhythm (Normal), other rhythms (Other), atrial fibrillation (AF), and noisy signals (Noisy).

**Figure 1:**
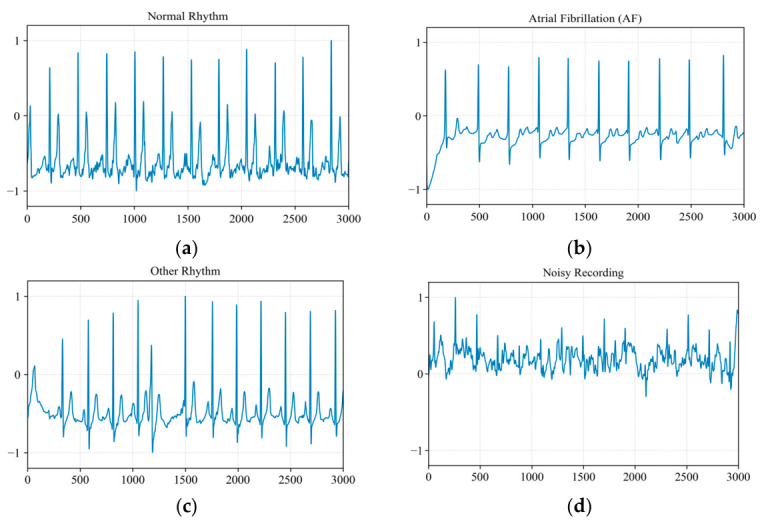
Representative ECG waveforms from the PhysioNet 2017 dataset across different categories: (**a**) Regular sinus rhythm recording, (**b**) AF signal recording, (**c**) Other rhythm recording, and (**d**) Noisy signal recording.

Figure 1 illustrates representative waveforms of the four distinct types of ECG recordings. Due to the long-tailed distribution characteristic of real-world clinical data, the number of Normal class samples (5154) far exceeds that of the AF class (771). This pronounced class imbalance can easily lead to a decrease in the model’s capability of recognizing the minority class (AF). Additionally, the proposed model’s adaptability was also evaluated on the MIT-BIH Arrhythmia dataset for training and evaluation [16].

### 3.2. Wearable ECG Signal Refinement

To boost the effectiveness of extracting features from ECG data, we performed necessary preprocessing on the raw ECG recordings. First, to eliminate baseline drift and low-frequency motion artifacts commonly encountered in wearable devices, we applied a fourth-order Butterworth high-pass filter with a cutoff frequency of 1 Hz using zero-phase filtering to preserve QRS phase characteristics [17]. Second, to account for amplitude variations across different subjects and recording environments, Z-score normalization (zero mean, unit variance) was applied to each segment to facilitate faster model convergence and improve training stability [18]. No low-pass filter was applied to avoid attenuating high-frequency components critical for arrhythmia detection.

For the Butterworth high-pass filter, the transfer function is given by:

(1) $$H\left(s\right)=\frac{s^{2n}}{s^{2n}+\left(\frac{\omega_{c}}{Q}\right)s^{n}+\omega_{c}^{2n}}$$

where $\omega_{c}$ denotes the cutoff frequency and n represents the filter order.

The Z-score normalization can be expressed as:

(2) $$z=\frac{x-\mu }{\sigma +\varepsilon }$$

where μ denotes the signal mean, σ represents the standard deviation, and ε is a small constant introduced to prevent division by zero.

### 3.3. Generation of Synthetic ECG Samples via QA-GAN

The proposed QA-GAN comprises three key components: a spatio-temporal hybrid generator architecture, a kurtosis-based quality assessment mechanism, and a dynamic soft-label training strategy. Specifically, we introduce a statistical prior, i.e., the kurtosis of a signal, to assess signal quality [19], and employs a dynamic soft-label mechanism to reduce the weight of noisy samples rather than discarding data directly. This strategy effectively guides the generator to prioritize learning high-quality morphological features, thereby addressing data imbalance while improving the quality of synthesized samples.

#### 3.3.1. Kurtosis-Based Signal Quality Evaluation (KSQE)

Based on the heavy-tailed and sparse nature of ECG signals in the time domain, this study adopts kurtosis as a statistical descriptor for quantifying signal quality. In statistics, kurtosis is used to measure the tail thickness or peakedness of a probability distribution. High-quality ECG signals exhibit significant sparsity in the time domain. They remain at the isoelectric line in most situations, with substantial voltage fluctuations occurring only during QRS complexes. This peaked, and heavy-tailed distribution manifests statistically as a very high kurtosis value. In contrast, Gaussian white noise or chaotic electromyographic artifacts typically exhibit a uniform distribution, characterized by relatively low kurtosis values. To verify the effectiveness of this statistical property in real-world scenarios, we calculated the kurtosis values of the representative AF and noisy samples shown in Figure 1. The kurtosis value (k = 19.81) of the high-quality AF signal in Figure 1b is significantly higher than that (k = 9.64) of the noisy signal in Figure 1d. This numerical difference demonstrates that the kurtosis of ECG signals can serve as a robust metric for distinguishing effective signals from background noise. Considering a segment of real ECG data, the kurtosis calculation is as follows:

(3) $$K\left(x\right)=\frac{E[(x-\mu {)}^{4}]}{\sigma^{4}}$$

where μ denotes the signal mean and σ represents the standard deviation. By calculating K(x) for each sample, we can quantify the prominence of QRS complexes within the signal, thereby distinguishing high-quality samples from noisy ones. This step provides a mathematical basis for subsequent soft label generation.

#### 3.3.2. The Architecture of QA-GAN

**Figure 2:**
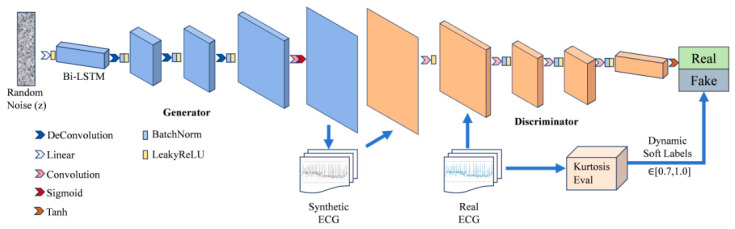
Diagram of the Quality-Aware Generative Adversarial Network (QA-GAN).

To simultaneously capture the global rhythm characteristics and local morphological details of ECG signals, we designed a hybrid generator architecture. As shown in Figure 2, the generator is conditioned on a random noise vector z, which serves as the input. It first employs a bidirectional LSTM (BiLSTM) [20] layer to characterize long-range temporal correlations and global rhythm patterns. Subsequently, the output of this module is fed into a series of one-dimensional transposed convolutional layers (ConvTranspose1d) for upsampling to reconstruct high-resolution waveform details such as QRS complexes. By employing the Tanh activation function, the final output is restricted within [−1, 1]. The discriminator adopts a standard CNN architecture to differentiate real signals from synthetic ones.

#### 3.3.3. Dynamic Soft-Label Training Strategy

QA-GAN retains the adversarial game mechanism of generative adversarial networks but redesigns the loss function to account for the characteristics of ECG signals. Traditional GANs typically assign a uniform hard label to all real samples, implicitly assuming that all training samples are of high quality. However, in clinical datasets such as PhysioNet 2017, real samples are often contaminated by baseline drift or artifacts. Forcing the discriminator to recognize such noisy samples as real leads the model to erroneously fit the noise distribution. To this end, as illustrated in Figure 2, we introduced a dynamic soft-label reweighting strategy [21], the target label is adaptively adjusted based on signal quality, thereby mitigating the misleading effect of low-quality samples on the training process. The target label y for a real sample x is no longer a constant, but is dynamically determined by its kurtosis value K(x):

(4) $$y_{real}=\alpha +(1-\alpha )\cdot tanh(\frac{log(K\left(x_{real}\right)+1}{5.0})$$

The mapping function ensures that y∈[α,1.0] signal contains clear QRS complexes (high kurtosis) approaches 1. When the signal-to-noise ratio is extremely low, such that QRS features are obscured, the target confidence is softened to the lower bound of α, thereby penalizing the discriminator from overfitting to low-quality samples. We redefine the discriminator’s loss function as:

(5) $$L_{D}=-E_{x\sim P_{data}}\left[y_{real}\cdot logD\left(x\right)\right]-E_{z\sim P_{z}}[\log\left(1-D\left(G\left(z\right)\right)\right)]$$

This strategy acts as a quality filter while training, effectively suppressing the generator’s tendency to learn the noise distribution inherent in the training set.

However, a limitation of relying solely on kurtosis is that sharp motion artifacts can occasionally exhibit high values, potentially misleading KSQE. In PEAC-Net, this is mitigated in two ways: first, KSQE utilizes continuous soft labels rather than binary decisions to minimize misjudgment impacts; second, sporadic artifacts rarely maintain high kurtosis across consecutive segments. To fully eliminate this vulnerability, future work will involve a parallel artifact discrimination module. This module will extract complementary morphological and spectral features, such as waveform skewness, peak duration, and low-frequency energy (0.5–5 Hz), to achieve robust multi-metric quality evaluation.

### 3.4. Proposed Model Architectures for AF Classification

**Figure 3:**
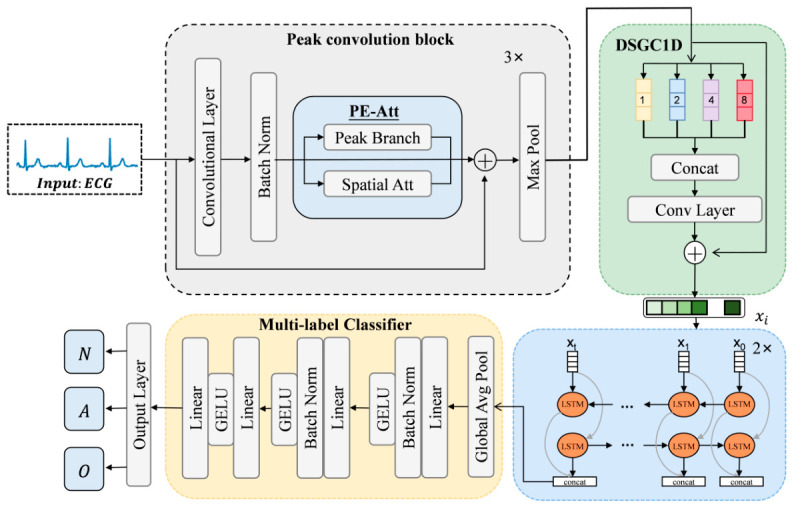
Diagram of the PEAC-Net network.

The PEAC-Net proposed in this study embodies the concept of multi-scale spatiotemporal decoupling: the front end focuses on local morphological transitions via PE-Att, while the backend models global long-range dependencies through DSGC1D and BiLSTM. The model, as depicted in Figure 3, is composed of three core modules: the Peak Convolution Block, DSGC1D, and BiLSTM. The input signal of the Peak Convolution Block is first passed through three stacked peak convolutional blocks, in which the Peak-Enhanced Attention Module (PE-Att) utilizes parallel peak branches and spatial attention branches to precisely localize and enhance the positions of R-waves and P-waves. Subsequently, the obtained feature maps are sent to the multi-scale dilated convolution module DSGC1D to conduct further processing. This multi-branch design constructs diversified receptive fields, aiming to achieve joint modeling of local waveform details and long-range patterns spanning multiple heartbeats. Finally, the feature sequence is fed into a bidirectional LSTM to model temporal dependencies between heartbeats, and the diagnostic result is output through a classifier. Table 2 provides a comprehensive description of the entire framework configuration of the model.

#### 3.4.1. Peak Convolution Block

**Figure 4:**
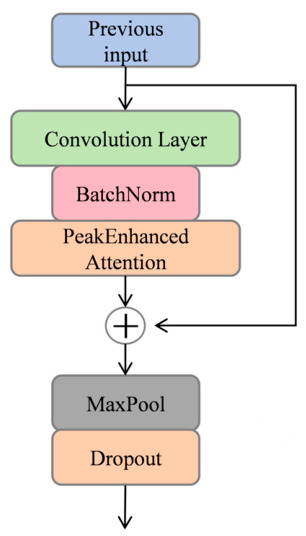
Diagram of the Peak Convolution Block.

The initial part of the proposed PEAC-Net model is mainly composed of three cascaded Peak Convolution Blocks (i.e., CB1, CB2, and CB3 in Table 2), which share the same architecture (as shown in Figure 4) but with different kernel configurations. In the Peak Convolution Block of PEAC-Net, the convolutional features obtained from the convolutional layer are fed into the PE-Att module for further feature enhancement. To preserve the original signal details and prevent gradient vanishing, the module introduces a residual connection, which directly adds the attention-weighted features to the convolutional features input to the module. Finally, after three cascaded convolution and pooling stages, the attention-enhanced feature sequence is fed into the subsequent DSGC1D module for further processing.

#### 3.4.2. Peak-Enhanced Attention Module

**Figure 5:**
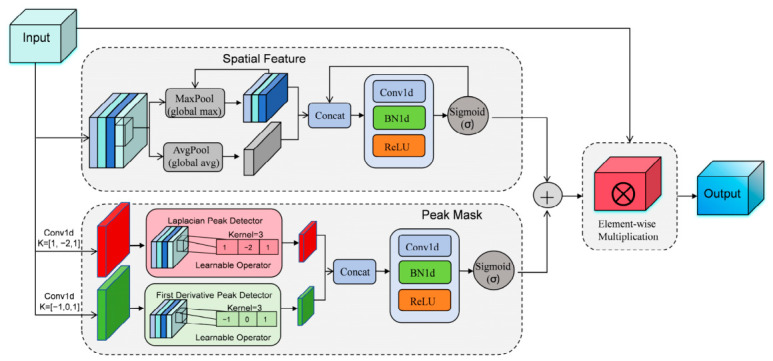
Diagram of the Peak-Enhanced Attention Module.

The Peak-Enhanced Attention (PE-Att) module is used to construct an adaptive feature enhancement mechanism aimed at suppressing interference from irrelevant background information by reinforcing critical temporal segments in ECG signals, such as abrupt changes in QRS complexes and inflection points of P/T waves. As illustrated in Figure 5, the Peak-Enhanced Attention (PE-Att) mechanism comprises a spatial attention branch [22] and a learnable peak mask generation branch connected in parallel. The latter integrates adaptive first-order and second-order derivative convolutional kernels, convolutional layers, and nonlinear activation functions.

The spatial attention branch, as depicted on the top side of Figure 5, operates by capturing spatial relationships among features to generate a corresponding attention map. To aggregate spatial features, we use average and max pooling along the channel dimension to produce two context descriptors: $F_{avg}^{S}$ and $F_{max}^{S}$. These two descriptors are combined by concatenation across the channel dimension and subsequently fed into a standard convolutional layer, yielding the spatial attention map $M_{S}\left(F\right)$. This process is mathematically expressed as follows:

(6) $$M_{S}\left(F\right)=\sigma \left(f^{v\times v}\left(\left[AvgPool\left(F\right);MaxPool\left(F\right)\right]\right)\right)=\sigma (f^{v\times v}\left[F_{avg}^{S};F_{max}^{S}\right])$$

where σ denotes the activation function of Sigmoid, and $f^{v\times v}$ denotes the convolution operation with a v×v kernel.

To further capture diagnostically significant morphological transitions in ECG signals, a peak enhancement branch is constructed. As shown in the Peak Mask branch of Figure 5, this branch employs a set of learnable derivative kernels to extract first-order and second-order variation features of the signal. One set corresponds to the discrete representation of the first-order derivative to capture rapid signal ascents [23]. The other set consists of Laplacian kernels that preserve symmetry to capture waveform inflection points [24]. Assuming the input feature is F, this branch first computes the first-order derivative features $D_{1}$ and second-order derivative features $D_{2}$ through two parallel convolutional layers. The extracted derivative features are then aggregated and subjected to a nonlinear transformation to generate the peak mask $M_{p}(F)$:

(7) $$M_{p}(F)=\sigma (f_{conv}(ReLU(f_{agg}(D_{1},D_{2}))))$$

where $f_{agg}$ denotes the feature aggregation operation, and $f_{conv}$ is the convolutional layer used for feature mapping. Equation (7) represents the mapping of the two types of derivative features into a peak mask via the Sigmoid function.

The final attention feature map is acquired by element-wise multiplication of the input features with the attention maps generated by the two branches, achieving weighted enhancement of key features and soft-threshold suppression of background noise.

#### 3.4.3. Dilated Separable Group Convolution

Although the front-end peak convolution blocks can effectively extract local morphological features, standard convolution operations are limited by fixed receptive fields and therefore insufficient for simultaneously capturing long-range rhythm patterns spanning multiple heartbeat cycles. To expand the receptive field and aggregate multi-scale context without significantly increasing computational complexity, we introduced a one-dimensional multi-scale dilated separable group convolution module (DSGC1D) after the cascaded convolutional layers.

**Figure 6:**
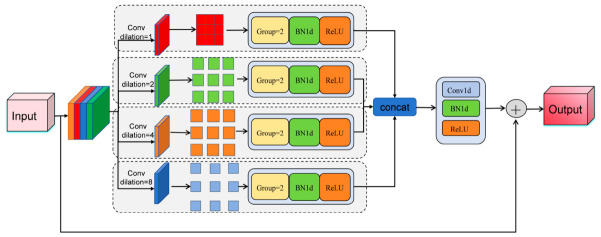
Architecture of the one-dimensional dilated separable group convolution (DSGC1D) module.

As shown in Figure 6, the DSGC1D module accepts the output generated by the peak-enhanced attention convolution module as its input. In the DSGC1D module, four parallel convolutional branches were employed to establish differentiated temporal receptive fields. Each branch is equipped with one-dimensional convolutional filters of the same kernel size, but different dilation rates. The introduction of different dilation rates allows the receptive field to grow exponentially while the parameter count being kept unchanged [25]. To reduce the number of parameters and improve computational efficiency, a grouped convolution strategy was also adopted in all branches [26]. Subsequently, a 1D convolutional layer employing a kernel of size 1 (Mixer) is applied for cross-channel information interaction and feature reconfiguration. Finally, to preserve salient features of the original signal and alleviate gradient vanishing, the module introduces a residual connection. After this cascaded branch aggregation and residual fusion process, the output feature sequence is fed into the subsequent bidirectional LSTM module for global temporal modeling and final classification.

## 4. Results

### 4.1. Experimental Setup

The hardware environment mainly consisted of an NVIDIA RTX 3060 (12 GB) GPU and an Intel i7-9700F CPU, and the software was built upon Python 3.11.4 and the PyTorch 2.1.0 deep learning framework. For data splitting, we first partitioned the dataset at the record level with an overall ratio of 8:2 for training and testing. Within the training portion, 15% of the training data was further reserved as a validation set. The Noisy category primarily consists of invalid or unreadable signals caused by electrode detachment or strong interference, and thus lacks pathological diagnostic significance. Therefore, to focus on evaluating the model’s discriminative ability for specific arrhythmias (Normal, AF, Other), we excluded Noisy class samples from the classification experiments. This way is consistent with many existing studies focused on arrhythmia classification. Training was conducted utilizing the Adam optimizer for parameter optimization, while the categorical cross-entropy functioned as the loss criterion. The learning rate was adjusted by monitoring the validation loss. The training configuration details of QA-GAN and PEAC-Net are presented in Table 3.

### 4.2. Data Augmentation Results

**Figure 7:**
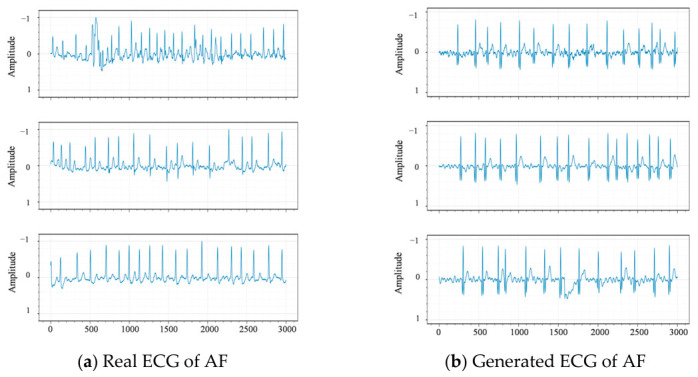
Comparison between synthetic AF signals generated by the proposed QA-GAN and real AF signals.

**Figure 8:**
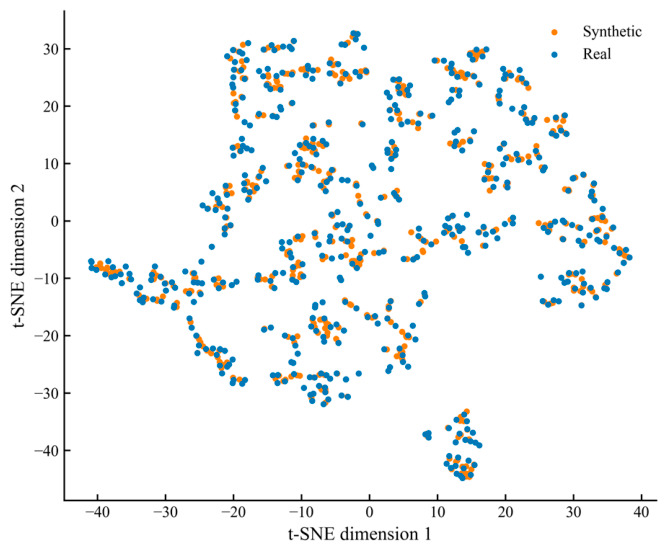
t-SNE comparison of real vs. QA-GAN-generated signal features.

As shown in Figure 7, we visually compared the synthetic AF signals generated by QA-GAN (Figure 7b) with real AF signals (Figure 7a). The visualization results demonstrate that the synthesized samples not only reproduce the typical rhythm irregularity of atrial fibrillation but also preserve clear QRS complex morphology. This result is consistent with expectations by introducing a quality-aware mechanism, namely, guiding the generator to focus on learning pathological features with high signal-to-noise ratios by suppressing the weight of low-quality samples. This indicates that QA-GAN is capable of generating signals with distinct pathological features, rather than simply fitting background noise. Furthermore, the t-SNE dimensionality reduction visualization in Figure 8 further confirms that the synthetic signals generated by QA-GAN exhibit a high degree of overlap and consistency with real AF signals in the feature distribution. This convincingly demonstrates that the quality-aware mechanism can guide the model to accurately capture the high-dimensional pathological features of atrial fibrillation and achieve comprehensive coverage of the real data manifold.

As shown in Table 4, the preprocessed training ECG signals were first segmented into 10 s slices, after which QA-GAN generated 4159 high-quality synthetic AF ECG signals. After QA-GAN augmentation, the count of AF samples in the training subset grew significantly from the original 617 (training subset) to a level comparable to that of the Other class, resulting in a relatively class-balanced training dataset.

### 4.3. Performance Metrics

To comprehensively quantify the performance of the classification network, this study adopts Precision, Sensitivity, Specificity, Accuracy, and F1-Score as evaluation metrics. The formulas are as follows:

(8) $$Acc=\frac{TP+TN}{TP+TN+FP+FN}$$

(9) $$Pre=\frac{TP}{TP+FP}$$

(10) $$Sen=\frac{TP}{TP+FN}$$

(11) $$Spe=\frac{TN}{FP+TN}$$

(12) $$F1\;score=2\times \frac{Pre\times Sen}{Pre+Sen}$$

TN, TP, FN, and FP represent the counts of true negative, true positive, false negative, and false positive predictions, respectively. Precision is then derived as TP/(TP + FP), quantifying the accuracy of positive predictions. Sensitivity, defined as TP/(TP + FN), measures the model’s ability to identify actual positive instances. The F1 score, which combines Precision and Sensitivity via their harmonic mean, offers a single measure of classification effectiveness.

### 4.4. ECG Classification Performance

**Figure 9:**
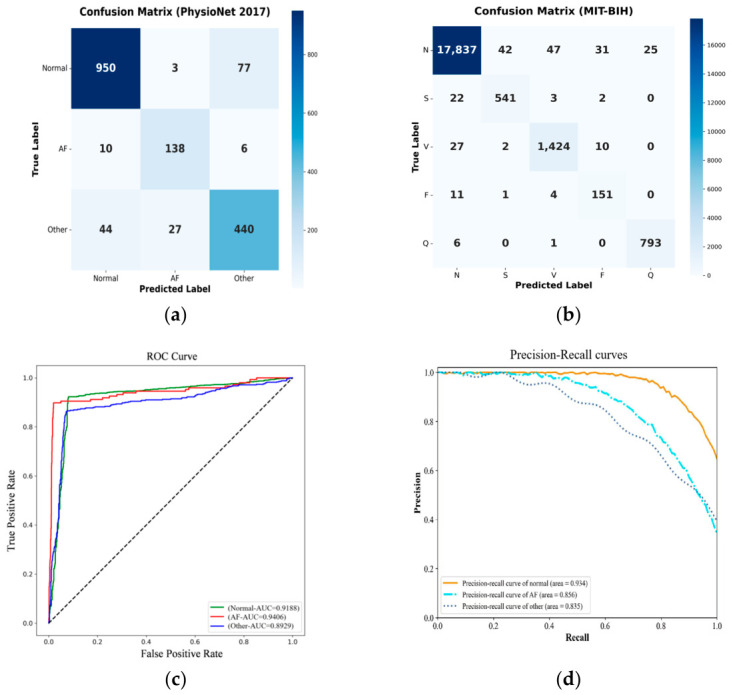
Performance summary of PEAC-Net: (**a**) confusion matrix on PhysioNet 2017, (**b**) confusion matrix on MIT-BIH, (**c**) ROC curves on PhysioNet 2017, (**d**) PR curves on PhysioNet 2017.

Figure 9a presents the confusion matrix results of PEAC-Net classification on the PhysioNet 2017 test set. Experimental findings reveal that our model yields comparable performance when applied to multi-class classification. As shown in Table 5, the model produced an accuracy of 0.902 and a macro F1-score of 0.880 on the test set, demonstrating the robustness and superiority of PEAC-Net in handling complex arrhythmia classification tasks. The confusion matrix results listed in Figure 9a indicate that PEAC-Net demonstrates strong performance in distinguishing Normal, AF, and Other rhythms on the PhysioNet 2017 dataset. Our model exhibits high precision in identifying Normal rhythm, with a sensitivity of 92.2% and a misclassification rate of only 0.3% into AF. Similarly, the proposed model demonstrates strong robustness in detecting AF, achieving a sensitivity of 89.6% with a misclassification rate of 10.4% (primarily misclassified as Normal). However, challenges arose in classifying Other rhythms, with a true positive rate of 86.1% but a relatively high misclassification rate. Specifically, 8.6% of Other samples were misclassified as Normal. This is possibly due to the heterogeneous property of the Other category in the PhysioNet database, which encompasses various non-specific arrhythmias that exhibit morphological similarities to normal sinus rhythm. The misclassification rate of Normal as Other (7.5%) and Other as Normal (8.6%) indicates a certain degree of mutual misclassification between Normal and Other rhythms. Similarly, the misclassification rates of AF as Other (3.9%) and Other as AF (5.3%) indicate a certain degree of feature confusion between these two rhythm categories. As illustrated in Figure 9c, the ROC curves for all three classes (Normal, AF, Other) closely approach the top-left corner, with the respective AUC values being 0.918, 0.941, and 0.893. The Precision-Recall curves in Figure 9d further confirm these results, with Average Precision values of 0.934 (Normal), 0.856 (AF), and 0.835 (Other). In summary, the overall performance of PEAC-Net demonstrates that the model is effective for accurate multi-class rhythm classification in single-lead ECG signals.

To further demonstrate the robustness and generalizability of our architecture, experiments were conducted on the PEAC-Net framework using another extensively utilized benchmark dataset—the MIT-BIH database. We first adopted an 80/20 random split protocol. The confusion matrix and accuracy results, as shown in Figure 9b and Table 6, demonstrate that the model achieved a macro F1-score of 0.943 and an accuracy of 0.988 on the MIT-BIH dataset. These experimental results indicate that PEAC-Net not only performs excellently on the PhysioNet 2017 dataset, but also maintains excellent performance on the MIT-BIH dataset after adapting to different lead configurations and sampling environments. In addition to this intra-patient evaluation, we further validated the framework under the more challenging inter-patient DS1/DS2 split protocol to assess its capability on unseen subjects; these complementary cross-patient results are documented in Tables S1 and S2 of the Supplementary Material.

### 4.5. Comparison with Similar Studies

The proposed PEAC-Net was further evaluated against other state-of-the-art approaches, and the results are listed in Table 7. Several deep learning approaches that demonstrated outstanding validation performance on the PhysioNet 2017 dataset were selected as benchmark models for comparison. These models are briefly described as follows:

1. Hong et al. adopted a deep neural network (DNN) for feature extraction from single-lead signals and then utilized an ensemble classifier for AF detection [27].
2. Yu et al. designed a cascaded architecture comprising three stages: first, denoising the raw ECG signals and calculating the heart rate (HR); subsequently, feeding the denoised signals together with HR values into convolutional layers for deep feature extraction; finally, performing classification through three fully connected layers [28].
3. Fang et al. proposed a one-dimensional CNN model named K-B2S, which extracts features using a feature extraction module consisting of five CNN blocks. The ultimate classification task is implemented via three fully connected layers together with a Softmax function to output probabilities for AF detection [29].
4. Kwon et al. first preprocessed the single-lead ECG signals from PhysioNet 2017 with the Pan-Tompkins algorithm (filtering and denoising), then generated spectrograms via short-time Fourier transform (STFT). These spectrograms were subsequently fed into pre-trained deep CNN models such as MobileNet, ResNet50, and DenseNet121, as well as a voting classifier that fused predictions from all three models, to achieve classification of cardiac rhythm categories, including AF [30].
5. Li et al. first performed signal inversion detection on the single-lead ECG signals of the PhysioNet 2017 and MIT-BIH, and then applied temporal mask decomposition to generate pseudo-QRS complexes and pseudo-T/P wave signals. The decomposed signals were subsequently fed into a residual neural network for AF detection [31].

The comparison results demonstrate that our PEAC-Net surpasses all other comparative methods with respect to all evaluation metrics. Specifically, it attains a macro F1-score of 0.88, whereas the best model among other approaches, proposed by Fang et al., yields an F1-score of 0.85. Our model achieves an F1-score of 0.86 for the AF class, outperforming all other methods listed in Table 6. These results demonstrate that the peak-enhanced attention mechanism effectively improves the recognition capability of AF-specific waveform patterns. Meanwhile, the F1-scores for Other and Normal classes are 0.85and 0.93, respectively, indicating the model’s balanced performance in multi-class classification tasks.

### 4.6. Noise Robustness Evaluation

To simulate real-world wearable ECG acquisition conditions, we added mixed noise to the test signals at varying signal-to-noise ratios (SNR). The mixed noise was generated by uniformly combining EMG noise and motion artifacts. EMG noise was simulated as band-limited Gaussian noise (20–100 Hz) to mimic high-frequency muscle interference, while motion artifacts were generated as low-frequency baseline drifts (0.5–2 Hz) and random walks to simulate body movement-induced disturbances. The noise amplitude was adjusted according to the target SNR. The classification results of various categories under different signal-to-noise ratios are presented in Table 8.

As shown in Table 8, under mixed noise conditions, the classification performance of all classes degrades to varying degrees, with the classes having fewer training samples being more severely affected. Specifically, under severe noise (0 dB), the F1 score of the AF class drops from 0.857 to 0.795, and the F1 score of the Other class drops from 0.851 to 0.780. Therefore, when facing severe noise interference, classes with fewer samples suffer significantly worse classification performance than classes with abundant samples.

## 5. Comparison and Ablation Experiments

To comprehensively validate the efficacy of the proposed constituent module in the PEAC-Net architecture, a series of experiments was conducted on the PhysioNet 2017 dataset, with configurations incrementally modified. Table 9 compares different attention mechanisms, and Table 10 evaluates various data augmentation strategies. The results are presented in Table 11, where the baseline model is progressively enhanced from a simple baseline configuration to the full architecture. The CNN-BiLSTM-based baseline model primarily relies on standard convolutional layers for local feature extraction and utilizes BiLSTM to capture temporal dependencies. As indicated by the experimental results, the baseline model attains comparatively low performance across all evaluation metrics, such as an F1-score of 0.793 and an accuracy of 0.830.

### 5.1. Integration of Multi-Scale Context

**Figure 10:**
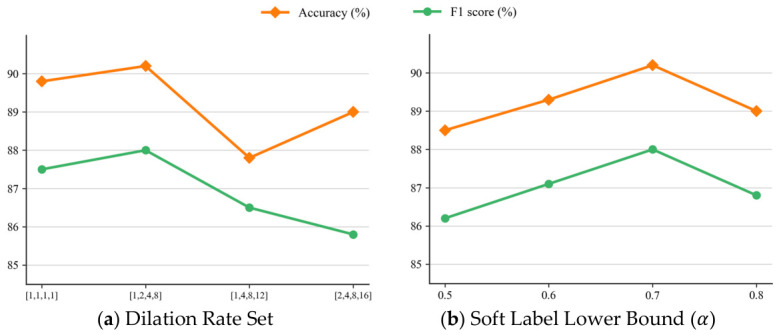
Ablation study of DSGC1D dilation rates and QA-GAN soft-label lower bound on the PhysioNet 2017 dataset. (**a**) Accuracy and Macro F1 under different dilation rate combinations. (**b**) Accuracy and Macro F1 under different soft-label lower bound values (LB).

After introducing the DSGC1D module into the baseline model, the performance of the model was boosted. As shown in Table 11 (Config 3), the F1-score improved to 0.815, and the Accuracy metrics increased to 0.863. As illustrated in Figure 10a, the dilation rate combination [1,2,4,8] achieves the highest accuracy and F1-score among the evaluated configurations. Although adding DSGC1D slightly increases model complexity, it yields significant performance gains through the parallel configuration of different dilation rates. This configuration enables the model to overcome the limitations of fixed receptive fields, simultaneously capturing local waveform details and long-range rhythm dependencies. Furthermore, through multi-scale feature fusion, it helps reduce overfitting to single-scale features, thereby enabling more comprehensive attention to relevant temporal patterns in the ECG data.

### 5.2. Comparison of Attention Mechanisms

We further compare the proposed PE-Attention with existing attention mechanisms under the same baseline setting (without data augmentation). As presented in Table 9, SE-Attention achieves an accuracy of 0.849 and an F1-score of 0.799, while Spatial Attention improves the performance to 0.864 and 0.821, respectively. Our PE-Attention achieves the best results among all attention mechanisms, reaching an accuracy of 0.877 and an F1-score of 0.835, outperforming Spatial Attention by 0.013 and 0.014, respectively. PE-Att introduces learnable derivative kernels and more computational paths. By considering ECG signal characteristics, it provides parallel feature extraction capability specifically targeted at signal transition points, thereby overcoming the limitation of weight dispersion inherent in traditional attention mechanisms. This property reduces the model’s attention to baseline waveforms and enables more precise capture of critical pathological features.

### 5.3. Impact of Data Augmentation Strategy

The results in Table 10 demonstrate the effectiveness of different data augmentation methods. Compared to the baseline without augmentation (F1 = 0.793), SMOTE improves the accuracy to 0.842. Random Oversampling (RO) achieves an accuracy of 0.836 and an F1-score of 0.799, while Class-weighted Loss (WL) achieves an accuracy of 0.848 and an F1-score of 0.809, both showing moderate improvements over the baseline. Our proposed QA-GAN further enhances the performance, achieving an accuracy of 0.858 and an F1-score of 0.816. As shown in Figure 10b, the soft-label lower bound of 0.7 yields the highest accuracy and F1-score among the candidate values. When integrated into the full PEAC-Net architecture, QA-GAN contributes to the highest overall performance, as shown in Table 11 (Config 7). Therefore, by using a kurtosis-driven soft-label mechanism to prevent noise fitting, QA-GAN can effectively alleviate the long-tail distribution problem by generating high-quality synthetic samples.

### 5.4. Model Complexity Analysis

Table 12 summarizes the computational complexity of PEAC-Net. The model contains 995,461 trainable parameters and requires 0.81 GFLOPs for processing a 10 s ECG segment. The average single-sample inference time is 6.82 ms, demonstrating its suitability for real-time wearable applications.

## 6. Visualization Analysis

To verify whether PEAC-Net exhibits interpretability comparable to clinical diagnosis in single-lead ECG analysis, we conducted a series of visualization experiments. By analyzing attention weight heatmaps and the evolution of adaptive derivative kernels, we revealed the model’s capability to focus on distinct morphological waveforms within ECG signals.

### 6.1. Visualization of Attention Mechanism

**Figure 11:**
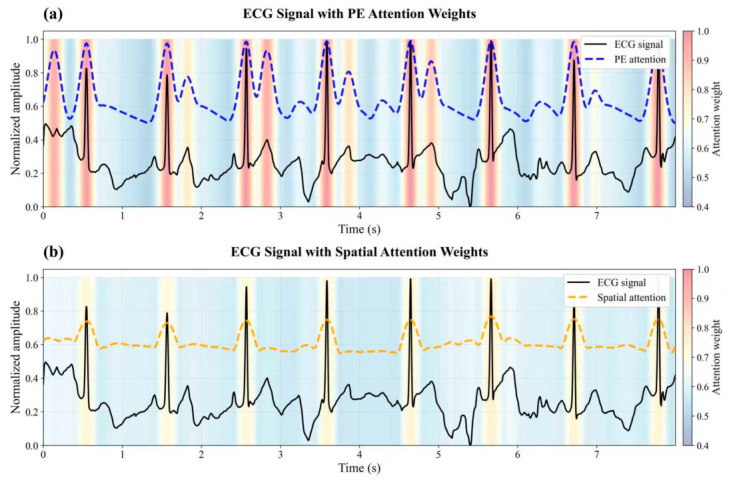
Comparison of attention weight heatmaps between two attention mechanisms on normal rhythm samples: (**a**) PE-Attention, (**b**) Spatial Attention.

**Figure 12:**
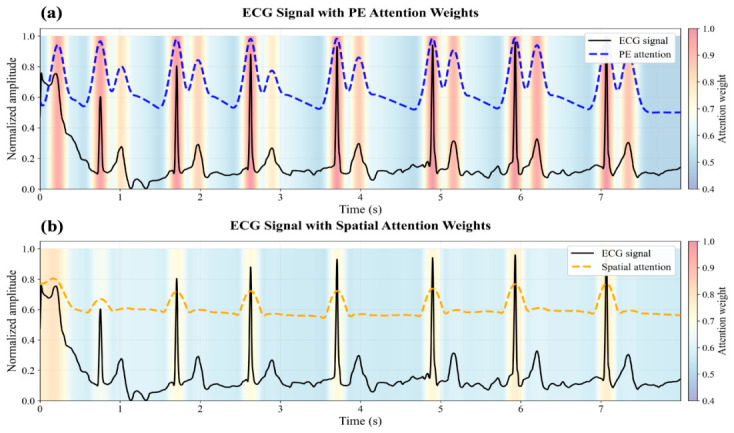
Comparison of attention weight heatmaps between two attention mechanisms on atrial fibrillation rhythm samples: (**a**) PE-Attention, (**b**) Spatial Attention.

We selected two representative rhythm samples (atrial fibrillation and normal sinus rhythm) to compare the weight distribution differences between the proposed Peak-Enhanced Attention (PE-Att) and traditional Spatial Attention (Spatial-Att). In Figure 11 and Figure 12, each row corresponds to the attention weight distributions of two different mechanisms for the same sample. The black line denotes the original ECG waveform, while the dashed line indicates the attention weight heatmap. A darker color in the heatmap signifies a higher degree of focus by the model on that specific region.

As shown in Figure 11 and Figure 12, the weight distributions of the two attention mechanisms exhibit significant differences. Figure 11 illustrates the weight distribution for a normal sinus rhythm sample. As shown in Figure 11a, PE-Att exhibits highly selective attention, with its attention weights rapidly rising to a high-value range of [0.9, 1.0] at the QRS complex locations, while rapidly decreasing and maintaining a relatively low level of 0.5 to 0.6 across the more quiescent baseline regions of the ECG waveform. This substantial variation in weight distribution indicates that the model can effectively suppress interference from background information and better focus on diagnostically significant R-peaks. In contrast, although the weight curve of Spatial-Att in Figure 11b also exhibits periodic fluctuations, its peak values only reach approximately 0.75, and the weights remain consistently around 0.5 throughout the quiescent baseline regions. This narrow fluctuation range indicates that Spatial-Att is less capable than PE-Att in accurately localizing key ECG waveforms.

Similarly, for the AF sample in Figure 12a, PE-Att also exhibits high-contrast characteristics, with its weight values varying across a wide range of [0.5, 1.0]. Particularly at the R-peak locations, the weight response approximates 1.0, exceeding the maximum value of approximately 0.75 observed in Spatial-Att as shown in Figure 12b. This numerically confirms that the PE-Att module possesses superior localization capability for dominant rhythm points. Furthermore, for the T-wave immediately following the QRS complex, PE-Att exhibits a distinct secondary peak in the weight curve, with values rising to approximately 0.8, accurately delineating the morphological boundaries of AF characteristics. In contrast, the weight curve of Spatial-Att in this region merely shows a gradual decline following the R-wave response, failing to form a distinct focal point for this feature.

### 6.2. Visualization of Derivative Kernel Evolution

**Figure 13:**
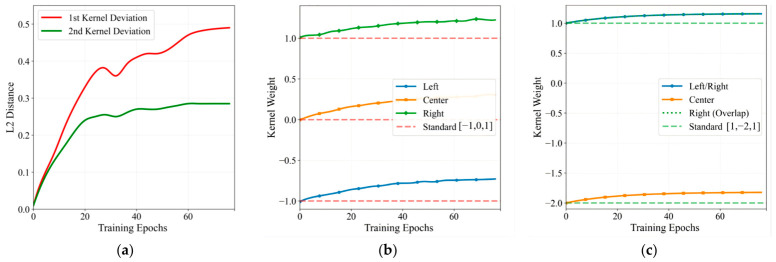
Evolution of learnable derivative kernel parameters during training. (**a**) L2 distance between current kernel parameters and their initial values across training epochs; (**b**) evolution of the three parameters of the first-order derivative kernel; (**c**) evolution of the three parameters of the second-order derivative kernel.

To verify the adaptive capability of the learnable derivative kernels, we visualized the evolution trajectory of the kernel parameters during training (Figure 13). The first-order kernel evolves from the initial [−1, 0, 1] to an asymmetric form [−0.704, 0.323, 1.225]: the absolute weight on the left decreases while the weight on the right increases significantly, forming a difference operator that enhances the response to the rising edge of the R-wave. The second-order kernel evolves from [1, −2, 1] to [1.166, −1.821, 1.148]: the left and right weights increase synchronously while maintaining symmetry, and the absolute value of the center weight decreases, achieving smooth detection of P/T wave inflection points. The L2 distance increases rapidly during the early training stage and then stabilizes, indicating that the model has adaptively learned the optimal kernel parameters.

## 7. Limitations and Future Work

Although the framework proposed in this study, which combines QA-GAN data augmentation and PEAC-Net classification, demonstrates excellent performance in single-lead ECG diagnosis, it still has certain limitations that need to be further investigated in future work.

First, this study primarily relies on a single-lead ECG for rhythm classification. Although single-lead devices offer significant advantages in portability, they only provide a projection of cardiac electrical activity along a single vector compared to the clinical standard 12-lead ECG [33]. This implies that the model may struggle to capture complex pathological features requiring multi-view information, such as myocardial ischemia localization or certain conduction blocks, thereby posing a risk of overlooking potential complications. Current experimental validation is primarily based on the publicly available PhysioNet 2017 dataset. Although the model performs excellently on the homologous test set, its performance may degrade when confronted with entirely new data collected from different centers or devices due to a domain shift in data distribution. As noted in the study by Kachuee et al. [34], the transferability of deep learning models across different patient populations is often limited in the absence of targeted domain adaptation training. Furthermore, in the stricter DS1/DS2 patient-level split evaluation [35], the model showed poor performance on minority classes, highlighting the need for improved recognition of rare arrhythmias under inter-patient settings. Validation on more recent public datasets (e.g., CPSC 2018, PTB-XL) has not yet been conducted, which limits the generalizability claims of our model. In the future, we plan to address the domain adaptation problem to further strengthen the generalization ability of the proposed model, and we will further explore feature disentanglement mechanisms to extract category-invariant features for domain generalization across different acquisition devices [36].

Although the QA-GAN employed in this study effectively alleviates data imbalance, GANs are prone to mode collapse during training, which limits the diversity of generated samples. Moreover, the model’s robustness against out-of-distribution (OOD) noise or completely unknown arrhythmias has not been systematically evaluated. Following recent advances in OOD data augmentation [37], we plan to explore OOD augmentation strategies to enhance the model’s robustness in real-world deployment scenarios. In light of the significant advancements of diffusion models in the field of generative AI, future work will explore ECG generation methods based on diffusion probabilistic models. Relative to GANs, diffusion models are poised to synthesize ECG signals exhibiting higher fidelity and richer morphological diversity, thereby potentially augmenting the classifier’s capacity to identify infrequent arrhythmias. Another important direction is handling open-set recognition in real-world deployment, where completely new arrhythmia types not present in the training set may appear. Drawing inspiration from prompt-guided disentanglement frameworks for cross-domain fault diagnosis with class mismatch [38], we will investigate open-set recognition techniques to enable the model to identify unknown arrhythmia classes. Furthermore, the PE-Att module is inherently designed to emphasize R-peak regions, so its performance inevitably degrades when R-peaks are unclear or corrupted by noise. As shown in Section 4.6, this degradation is more severe for the AF and Other classes due to their smaller sample sizes, and is further exacerbated by the high-frequency noise amplification of the differential operators.

To achieve truly real-time home monitoring, the models must be deployable on resource-constrained wearable devices such as fitness bands or smart watches. The current PEAC-Net incorporates a bidirectional LSTM and an enhanced attention module, leading to a considerable amount of model parameters and huge computational burdens. In the future, we will consider the lightweight design of the proposed model, using model pruning, weight quantization, and knowledge distillation techniques to cut the model’s computational cost and memory usage while maintaining high accuracy, so that it can be deployed on edge computing platforms with limited computational capacity. Additionally, we plan to design an R-peak quality screening module to assess R-peak clarity before classification, as well as a motion artifact detection module to identify and down-weight artifact-contaminated segments, allowing the model to rely more on other morphological features (P-wave, T-wave) or trigger a rejection mechanism when signal quality is poor.

## 8. Conclusions

This paper proposes an atrial fibrillation detection framework for single-lead ECG signals, aimed at achieving precise capture of subtle pathological features in single-lead recordings and alleviating the significant class imbalance problem attributable to the insufficiency of pathological samples. The proposed framework comprises three key stages: preprocessing, QA-GAN data augmentation, and PEAC-Net classification. First, signal filtering and normalization are employed to improve signal quality. Second, QA-GAN is introduced to generate high-fidelity synthetic samples. Finally, the PEAC-Net model integrates peak-enhanced attention, multi-scale dilated convolution, and BiLSTM to extract both local waveform details and global rhythm features, thereby achieving ECG signal classification. Experimental results on the PhysioNet 2017 dataset demonstrate that the proposed model exhibits excellent classification robustness in processing single-lead ECG signals. This study confirms the potential of single-lead signals in conveying clinically diagnostic information and provides an effective model reference for mobile heart health monitoring systems.

## Figures and Tables

**Table 1:** Detailed Category Information of the PhysioNet Challenge 2017 Dataset.

Class	Counts	Length(s)			
		Max	Median	Min	Mean
Normal	5154	61.0	30	9.0	31.9
AF	771	60	30	10.0	31.6
Other rhythm	2557	60.9	30	9.1	34.1
Noisy	46	60	30	10.2	27.1
Total	8528	61.0	30	9.0	32.5

**Table 2:** Network configuration of the PEAC-Net.

Module	Layer/Block	Configuration Details
Input Data		10s-ECG Signal (Length: 3000, Channel: 1)
Peak Conv Block 1 (CB1)	Conv Layer	Conv11_32_1 ^1^
	Attention	PE-Att (Spatial k = 7, Peak k = 3) ^2^
	Pooling & Reg	MaxPool(2), Dropout(0.2), BatchNorm, ReLU
Peak Conv Block 2 (CB2)	Conv Layer	Conv11_64_1
	Attention	PE-Att (Spatial k = 7, Peak k = 3)
	Pooling & Reg	MaxPool(2), Dropout(0.3), BatchNorm, ReLU
Peak Conv Block 3 (CB3)	Conv Layer	Conv7_128_1
	Attention	PE-Att(Spatial k = 7, Peak k = 3)
	Pooling & Reg	MaxPool(2), Dropout(0.3), BatchNorm, ReLU
Multi-scale Fusion	DSGC1D Block	Dilations = [1, 2, 4, 8], Groups = 2, Residual Add
	Output Channel	256 (Expanded for LSTM input)
Temporal Modeling	Bi-LSTM Block	Bi-LSTM [Input: 256, Hidden: 128, Layers: 2]
	Dropout	Rate: 0.4
Classifier (FC)	Global Pooling	Global Average Pooling (GAP)
	Dense Layers	Dense [128, 64], BatchNorm, GELU
	Output Layer	Dense [n_classes]
	Activation	SoftMax()

^1^ ConvK_C_S: denotes Conv(kernel size)(channels)(stride). ^2^ PE-Att kernel sizes: Spatial k = 7: used for the spatial attention branch to capture local waveform context; Peak k = 3: used for the peak detection branch, containing first-order derivative kernel [−1, 0, 1] and second-order derivative kernel [1, −2, 1].

**Table 3:** Training configuration details of QA-GAN and PEAC-Net.

Parameter	QA-GAN	PEAC-Net
Optimizer	Adam	Adam
Epoch	500	80
Batch size	32	32
Learning rate	0.0001	0.001
Loss function	Generator: standard adversarial loss; Discriminator: dynamic soft-label loss (Equation (5))	Categorical cross entropy

**Table 4:** The number of training samples pre- and post-data augmentation.

Class	Total	Testing	Training	Training Segments (Before QA-GAN)	Training Segments (After QA-GAN)
Normal	5154	1030	4124	12,641	12,641
AF	771	154	617	1841	6000
Other	2557	511	2046	6612	6612
Total	8482	1695	6787	21,094	25,253

**Table 5:** Per-class performance of the model on the PhysioNet 2017 test set.

Class	Precision	Sensitivity	Specificity	Accuracy	F1-Score
Normal	0.946	0.922	0.918	0.921	0.934
AF	0.821	0.896	0.980	0.973	0.857
Other	0.841	0.861	0.929	0.909	0.851
Overall Performance	0.868	0.894	0.943	0.902	0.880

**Table 6:** Performance of PEAC-Net on the testing dataset of MIT-BIH.

Class	Precision	Sensitivity	Specificity	Accuracy	F1-Score
N	0.996	0.991	0.978	0.989	0.994
S	0.923	0.952	0.997	0.996	0.937
V	0.962	0.973	0.997	0.995	0.968
F	0.778	0.904	0.997	0.997	0.836
Q	0.969	0.991	0.998	0.998	0.980
Overall Performance	0.926	0.963	0.993	0.988	0.943

**Table 7:** F1-Scores of the proposed model and other state-of-the-art approaches on the PhysioNet 2017.

Dataset	Method	F1-Score			
		AF	Normal	Other	Overall
Hong et al. [27]	Ensembled XGBoost	0.85	0.92	0.74	0.84
Yu et al. [28]	synthetic signal-enhanced DDCNN	0.81	0.91	0.78	0.83
Fang et al. [29]	Large Kernel + Concatenation	0.84	0.92	0.80	0.85
Kwon et al. [30]	DenseNet121	0.81	0.92	0.74	0.82
Li et al. [31]	Signal Decomposition + Dilated Residual	0.82	0.92	0.80	0.84
Ours	QA-GAN + PEAC-Net	0.86	0.93	0.85	0.88

**Table 8:** Classification performance of PEAC-Net on the PhysioNet 2017 dataset under varying signal-to-noise ratios (0 dB, 5 dB, 10 dB).

Class	SNR Levels								
	0 dB			5 dB			10 dB		
	Precision	Sensitivity	F1-Score	Precision	Sensitivity	F1-Score	Precision	Sensitivity	F1-Score
Normal	0.902	0.885	0.893	0.928	0.910	0.919	0.938	0.917	0.928
AF	0.758	0.835	0.795	0.803	0.882	0.841	0.815	0.892	0.852
Other	0.768	0.792	0.780	0.819	0.843	0.831	0.831	0.853	0.842
Overall	0.809	0.837	0.823	0.844	0.878	0.864	0.861	0.887	0.874

**Table 9:** Comparison of Different Attention Mechanisms (All without data augmentation).

Attention Mechanism	Accuracy	Macro F1	Precision	Sensitivity
SE-Attention [32]	0.849	0.799	0.789	0.810
Spatial Attention [22]	0.864	0.821	0.816	0.826
PE-Attention (Ours)	0.877	0.835	0.831	0.840

**Table 10:** Comparison of Different Data Augmentation Methods.

Augmentation Method	Accuracy	Macro F1	Precision	Sensitivity
No augmentation	0.830	0.793	0.773	0.815
SMOTE [3]	0.842	0.804	0.789	0.819
Random Oversampling	0.836	0.799	0.785	0.816
Class-weighted Loss	0.848	0.809	0.793	0.826
QA-GAN (Ours)	0.858	0.816	0.795	0.837

**Table 11:** Ablation study results of models with different settings on the PhysioNet 2017 dataset.

Config	QA-GAN	DSGC1D	PE-Att	Accuracy	Macro F1	Precision	Sensitivity
1				0.830	0.793	0.773	0.815
2	✓			0.858	0.806	0.795	0.819
3		✓		0.863	0.815	0.805	0.826
4			✓	0.877	0.835	0.831	0.840
5	✓	✓		0.884	0.855	0.841	0.871
6	✓		✓	0.894	0.863	0.852	0.875
7		✓	✓	0.880	0.852	0.845	0.860
8	✓	✓	✓	0.902	0.880	0.868	0.894

**Table 12:** Computational complexity of PEAC-Net.

Model Details	Params	GFLOPs	Inference Time (ms)
PEAC-Net	995461	0.81	6.82

## Data Availability

The PhysioNet Challenge 2017 dataset and the MIT-BIH Arrhythmia database used in this study are both publicly available. The PhysioNet 2017 dataset is accessible at https://physionet.org/content/challenge-2017/1.0.0/ (accessed on 20 January 2025), and the MIT-BIH database is accessible at https://physionet.org/content/mitdb/1.0.0/ (accessed on 20 January 2025). No additional private data were collected or analyzed in this work.

## Supplementary Materials

The following supporting information can be downloaded at: https://www.mdpi.com/article/10.3390/s26123852/s1, Table S1: Confusion matrix of PEAC-Net on MIT-BIH (DS2); Table S2: Performance of PEAC-Net on MIT-BIH (DS2).
