# Supplementary material for: Single-Lead ECG Arrhythmia Classification Based on Peak-Enhanced Attention Network and Quality-Aware GAN Data Augmentation Framework
Source: Sensors (Basel). 2026 Jun 17;26(12):3852. doi: 10.3390/s26123852 (PMC13307092; doi:10.3390/s26123852)
Supplement: Supplementary file 1 [file sensors-26-03852-s001.zip › sensors-4342966-supplementary.pdf]

Table S1 Confusion matrix of PEAC-Net on MIT-BIH (DS2)

	Class	**Predicted class**			
	type	N	S	V	F
**True**	N	43070	1444	231	267
**class**	S	380	1452	5	0
	V	134	239	2964	49
	F	318	0	18	52

Table S2 Performance of PEAC-Net on MIT-BIH (DS2)

**Class**	**Precision**	**Sensitivity**	**F1-Score**
N	0.974	0.957	0.965
S	0.463	0.790	0.584
V	0.921	0.875	0.897
F	0.141	0.134	0.137
Overall	**Accuracy: 0.939**	**Macro F1: 0.647**	
